# Melioidosis Outbreak after Typhoon, Southern Taiwan

**DOI:** 10.3201/eid1306.060646

**Published:** 2007-06

**Authors:** Wen-Chien Ko, Bruno Man-Hon Cheung, Hung-Jen Tang, Hsin-I Shih, Yeu-Jun Lau, Li-Rong Wang, Yin-Ching Chuang

**Affiliations:** * National Cheng Kung University Medical College, Tainan, Taiwan, Republic of China; † National Cheng Kung University Hospital, Tainan, Taiwan, Republic of China; ‡ Tainan Municipal Hospital, Tainan, Taiwan, Republic of China; § Chi Mei Medical Center, Tainan, Taiwan, Republic of China; ¶ Show Chwan Memorial Hospital, Changhua, Taiwan, Republic of China

**Keywords:** Melioidosis, Burkholderia pseudomallei, outbreak, southern Taiwan, typhoon, dispatch

## Abstract

From July through September 2005, shortly after a typhoon, 40 cases of *Burkholderia pseudomallei* infection (melioidosis) were identified in southern Taiwan. Two genotypes that had been present in 2000 were identified by pulsed-field gel electrophoresis. Such a case cluster confirms that melioidosis is endemic to Taiwan.

Melioidosis is an infectious disease caused by *Burkholderia pseudomallei* and is found in northern Australia and tropical countries in Southeast Asia (*1*). The incidence of melioidosis cases increases during the rainy season (*2*). Melioidosis often affects adults who have chronic underlying diseases, especially diabetes mellitus, and is often associated with illness and death. The mortality rate varies geographically, ranging from 19% in northern Australia to 68% in northeastern Thailand (*1*).

The first case of melioidosis in Taiwan, acquired in the Philippines, was reported in 1985 (*3*); since then several sporadic cases have been reported. In a recent comprehensive review, Taiwan was categorized as an area in which melioidosis may be endemic (*1*). However, this conclusion was deduced from a limited number of clinical cases reported in the literature. A total of 13 cases have been reported in Taiwan in patients who never traveled to melioidosis-endemic areas (*4*–*12*). These patients likely have indigenous cases of melioidosis, and no common source of *B*. *pseudomallei* isolates has been identified. We report a cluster of 40 cases of melioidosis after a typhoon hit Taiwan, which confirms that melioidosis is endemic in this country.

## The Study

Heavy rains from Typhoon Haitang on July 16, 2005, caused mudslides and flooding in central and southern Taiwan. The first clinical isolate of *B*. *pseudomallei* was found on July 29, 2005. Demographic and clinical data for melioidosis case-patients at 3 hospitals were collected.

Clinical information was collected on a case record form. For each patient, demographic data, including location, clinical signs, underlying illness, laboratory data, radiologic images, antimicrobial drug therapy, and clinical outcome, were obtained from medical records. Information about patients’ functional levels, recent exposure to mud or water before admission, and prior travel to Southeast Asia or Australia was obtained by telephone from the patients or their families if such information was incomplete or not available on medical charts. The study was reviewed and approved by the Institutional Review Board of Chi-Mei Foundation Medical Center.

Forty oxidase-positive, nonfermentative, gram-negative bacilli grew in Ashdown selective medium and showed characteristic dry, rough, blue-purple colonies. These isolates were then tested for flagellin genes by PCR. The paired primers used for PCR amplification were PMA-1 (5′-CTG TCG TCG ACG GCC GT-3′) and PMA-2 (5′-GGT TCG AGA CCG TTT GCG-3′) (*13*). The amplicons, ≈190 bp, were sequenced by using an ABI 3730 DNA Analyzer (Applied Biosystems, Foster City, CA, USA). These isolates showed 99% identity with the homologous region of *B*. *pseudomallei* ATCC23343 in the GenBank database.

In addition to the 40 isolates obtained in 2005, a total of 14 *B*. *pseudomallei* isolates from 14 sporadic cases were available for molecular typing, including 1 isolate found in 2000, 4 in 2001, 4 in 2002, 1 in 2003, and 4 in 2004. Only the first isolate from each case was studied. Two genetically distinct *B*. *pseudomallei* strains isolated in northern Taiwan (*9*) were used as reference strains. For pulsed-field gel electrophoresis (PFGE), bacterial plugs were digested with restriction endonucleases *Xba*I and *Spe*I. Digests were subjected to gel electrophoresis using the Chef Mapper System (Bio-Rad Laboratories, Hercules, CA, USA) with a bacteriophage λ DNA ladder. Gels were stained with ethidium bromide, viewed under UV light, and analyzed by using the Molecular Analyst System (Bio-Rad Laboratories).

Of 40 patients, 30 (75%) were male. Their mean age was 64.6 years (range 38–87 years). A total of 37 (92.5%) patients never traveled abroad, and 28 (70%) denied recent contact with mud or dirty water before their illness. Because 3 patients could not walk, infection by cutaneous contact with contaminated dirt or water was less likely. Twenty-seven (67.5%) patients had an underlying debilitating illness, predominantly diabetes mellitus (20 patients).

The most common initial symptoms were fever (29 patients, 72.5%) and cough (13 patients, 32.5%). Relevant prodromes lasting <72 hours before admission were noted in 25 (62.5%) patients, and 13 (32.5%) patients visited hospitals within 24 hours after onset of the illness, which suggests acute illness. The earliest onset of symptoms related to melioidosis was July 20, 2005. This onset was 4 days after the arrival of Typhoon Haitang, which suggests an incubation period of 4 days. Most patients (30, 75%) had *B*. *pseudomallei* bacteremia, and 20 of these patients had concomitant pleuropulmonary infections. Of 10 (25%) patients without bacteremia, pulmonary infections remained the predominant foci in 6. Eight (20%) patients died during hospitalization.

**Figure 1:**
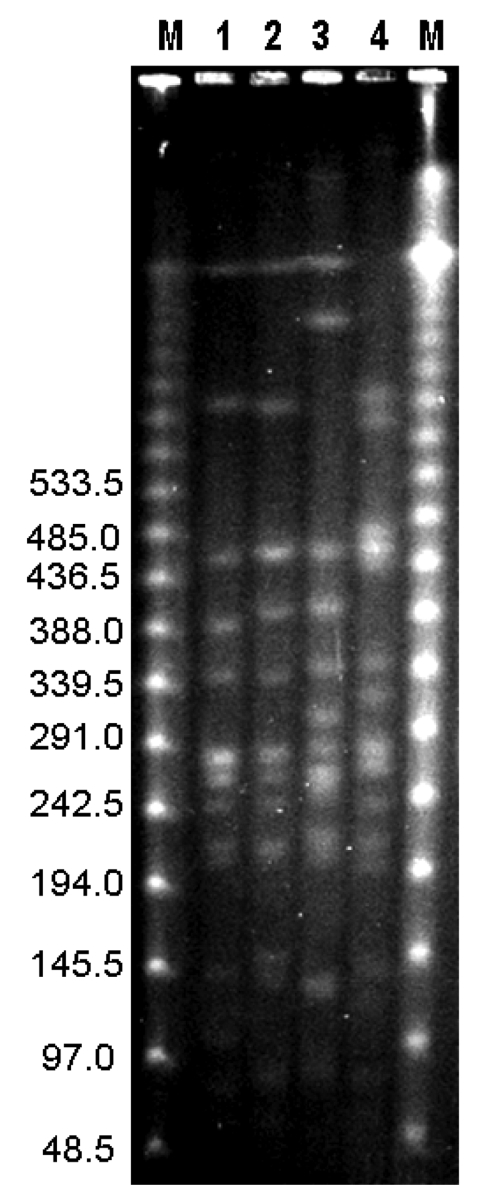
Pulsed-field gel electrophoresis of DNA from *Burkholderia pseudomallei* isolates digested with *Spe*I from patients with melioidosis in Taiwan. Lane M, bacteriophage λ DNA ladder (48.5 kb–970 kb). Lane 1, isolate from Kaohsiung County, 2005 (type A); lane 2, isolate from Tainan County, 2005 (type B); lane 3, isolate from northern Taiwan (type C); lane 4, isolate from northern Taiwan (type D). Values on the left are in kilobases.

**Figure 2:**
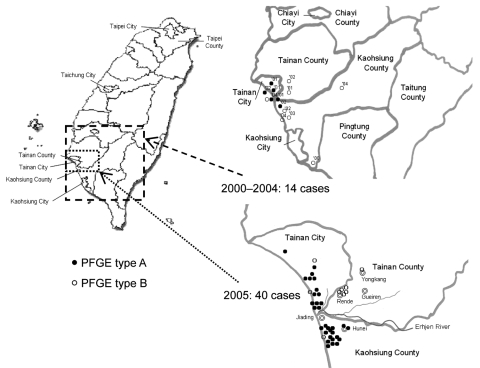
Geographic distribution of 14 sporadic cases of melioidosis, 2000–2004, and 40 clustered cases, 2005, Taiwan. Two pulsed-field gel electrophoresis (PFGE) genotypes (types A and B) of *Burkholderia pseudomallei* were present in southern Taiwan. The numbers in the upper right panel indicate year of isolation.

*Xba*I restriction profiles in PFGE provide a level of resolution similar to that obtained with multilocus sequence typing for *B. pseudomallei* isolates (*14*), but the discriminative sensitivity of *Spe*I was greater than that of *Xba*I in differentiation of our *B*. *pseudomallei* isolates. Thus, genotyping information obtained from PFGE *Spe*I profiles is useful in epidemiologic studies. Among the 54 isolates, 2 PFGE genotypes (types A and B) were identified in *Spe*I macrorestriction profiles (Figure 1). These genotypes were genetically distinct from the 2 reference isolates (types C and D). Genotypes A and B were found in isolates obtained as early as 2000 (Figure 2).

## Conclusions

Melioidosis became a reportable disease in Taiwan in 2000; through 2004, the Center for Disease Control in Taiwan received reports of 43 cases of *B*. *pseudomallei* infections (*15*). A total of 33 (77%) cases were reported in southern Taiwan. Increased rainfall in conjunction with Typhoon Haitang was observed from July through September 2005. At the end of September 2005, a total of 40 cases of melioidosis were identified. From October 2005 through March 2006, only 3 cases were identified, but these were not included in our study.

In conclusion, after widespread flooding caused by a typhoon, an outbreak of melioidosis occurred in southern Taiwan from July 2005 through September 2005. Two genotypic strains were found in clustered cases in 2005 and in sporadic cases found in Tainan and Kaohsiung counties in 2000. These findings confirm that environmental sources of *B*. *pseudomallei* are likely present in southern Taiwan and that melioidosis is endemic in this country.
